# Supplementary material for: Characterization of the Peri-Membrane Fluorescence Phenomenon Allowing the Detection of Urothelial Tumor Cells in Urine
Source: Cancers (Basel). 2022 Apr 26;14(9):2171. doi: 10.3390/cancers14092171 (PMC9105609; doi:10.3390/cancers14092171)
Supplement: Supplementary file 1 [file cancers-14-02171-s001.zip › supplementary materials and figures.pdf]

Supplemental Materials and Methods

Plasma membrane composition modulation assay

Miltefosine was produced by ApexBio Technology (Houston, USA) and purchased from Clinisciences (inc, Nanterre, France). Miltefosine was rehydrated in DMSO and stored at -80°C. Cells were seeded at a density of 40 000 cells/cm² 24hours before the treatment. Cells were treated with 8 to 12µM of miltefosine in serum-free media for 24h. Then cells were recovered and put in PreservCyt® for the staining or washed with PBS 3 times for cell death assay.

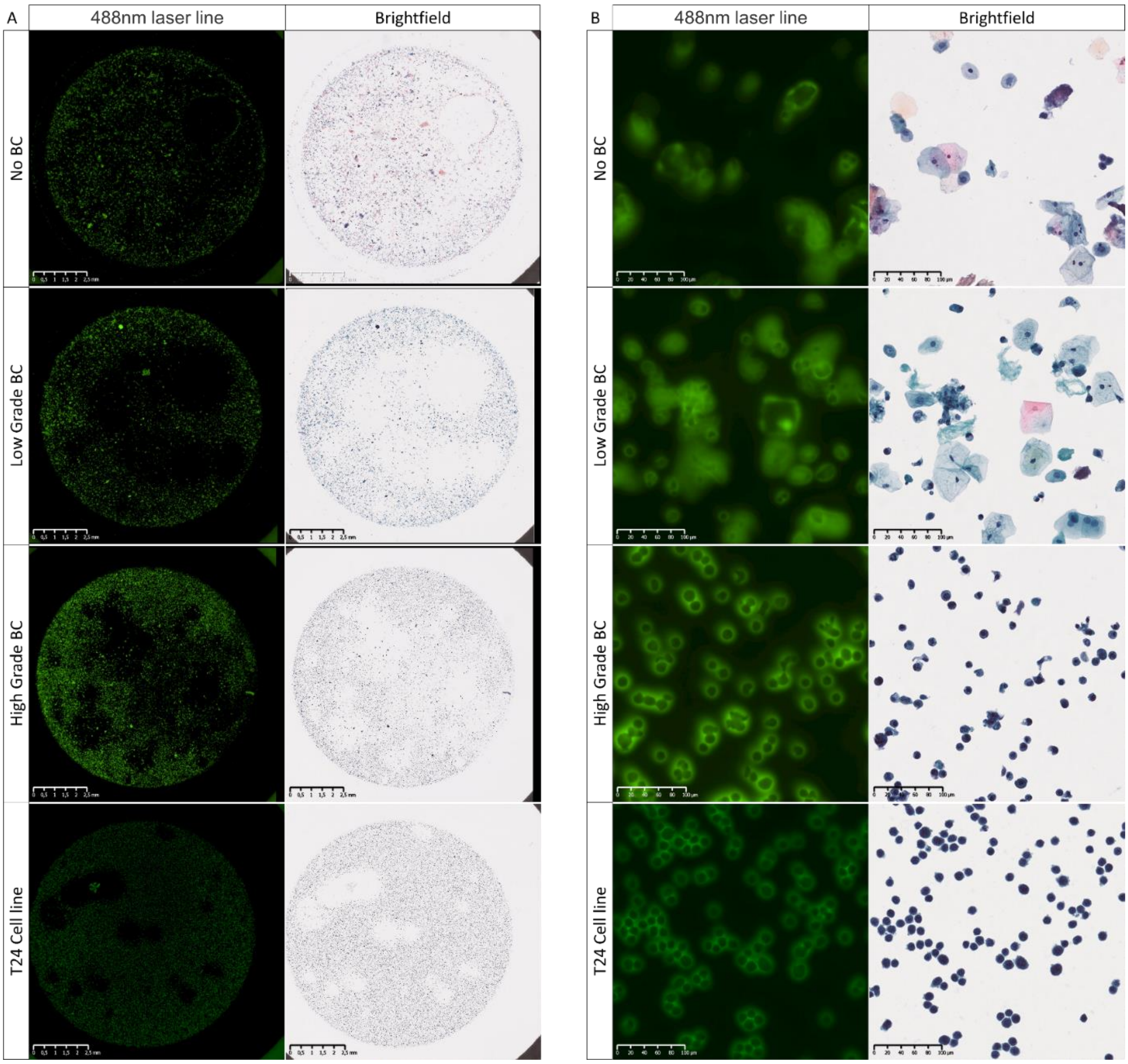

Figure S1. Characteristic of the fluorescence from healthy and tumoral urothelial cells. a) Global view of a urinary cytology acquired on the scanner of a healthy patient (No BC); a patient with LGBC (Low Grade BC); a patient with HGBC (High Grade BC) and T24 (T24 Cell line) in brightfield light and at 488nm**.** b) Zoomed view of a urinary cytology acquired on the scanner of a healthy patient (No BC); a patient with LGBC (Low Grade BC); a patient with HGBC (High Grade BC) and T24 (T24 Cell line) in brightfield light and at 488nm**.**

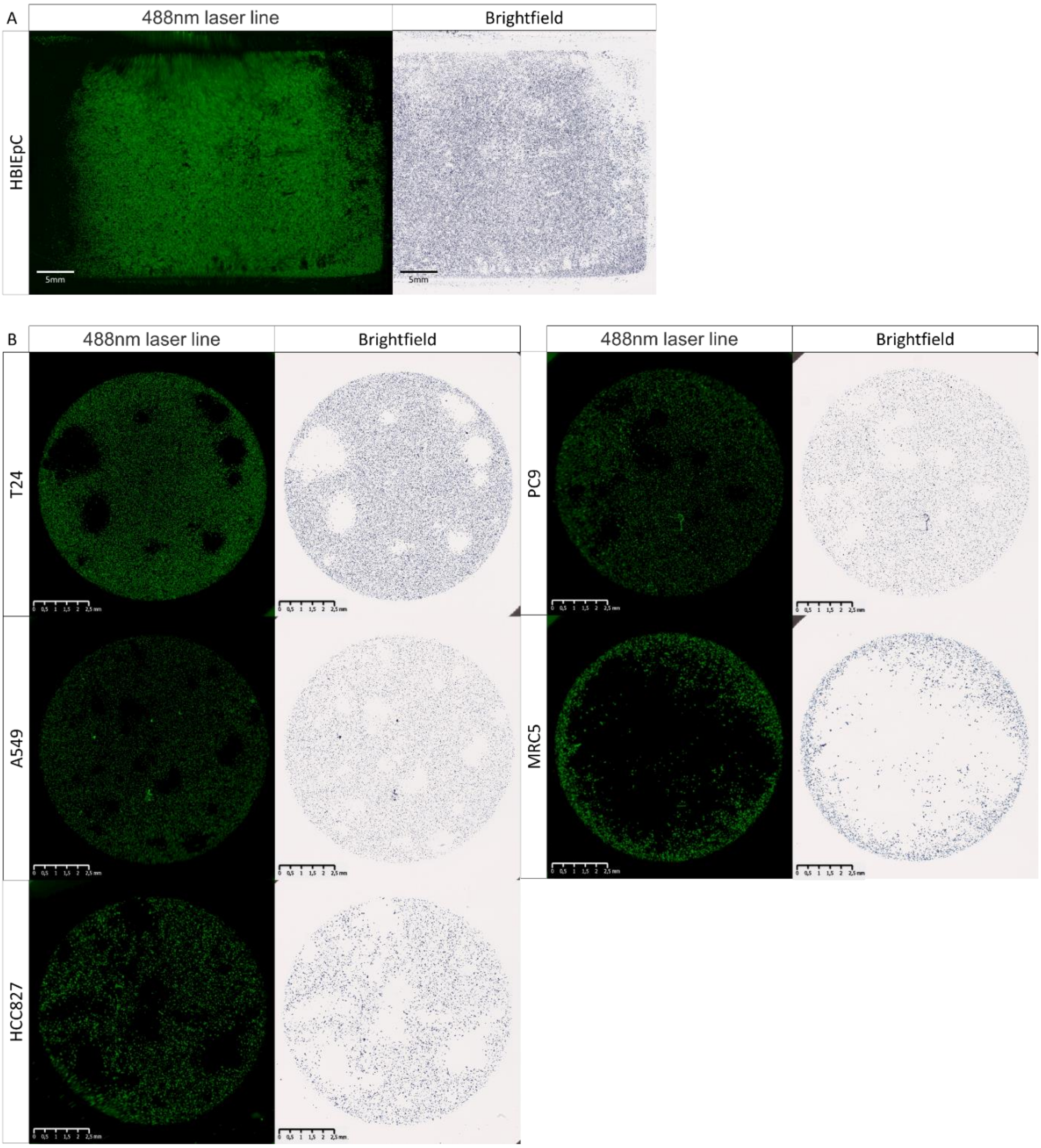

Figure S2. PMF on different cells line. a) Global view of a normal human primary bladder epithelial cell line acquired on the scanner. b) Global view cell lines acquired on the scanner.

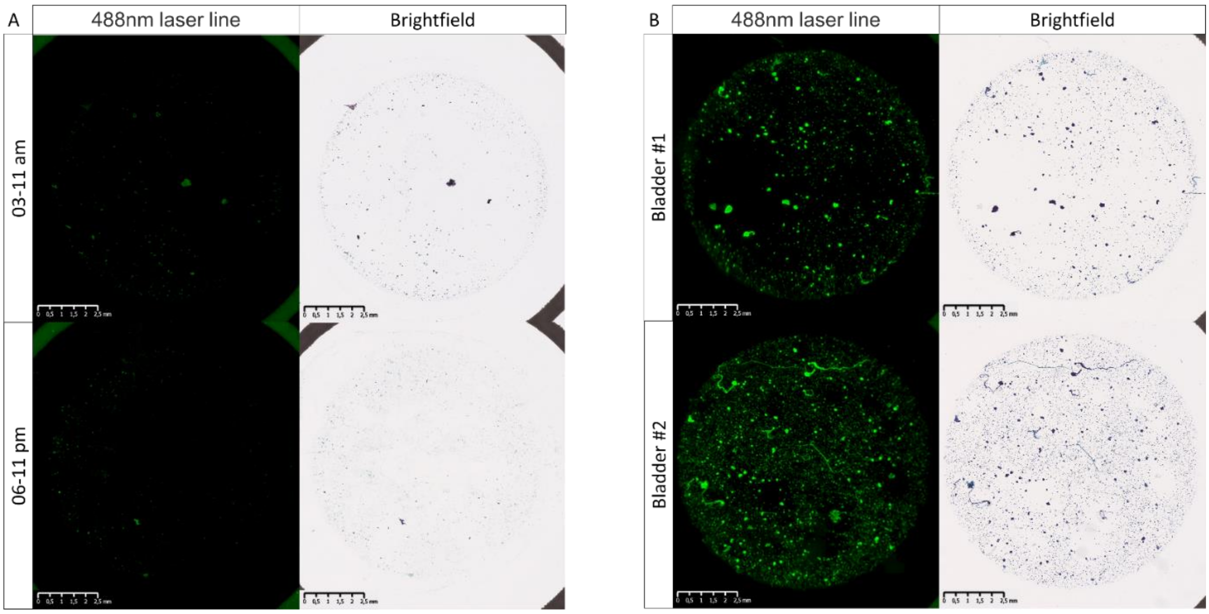

Figure S3. Fluorescence and PMF of UCU and UCUT. a) Global view of UCU acquired on the scanner. b) Global view of UCUT acquired on the scanner.

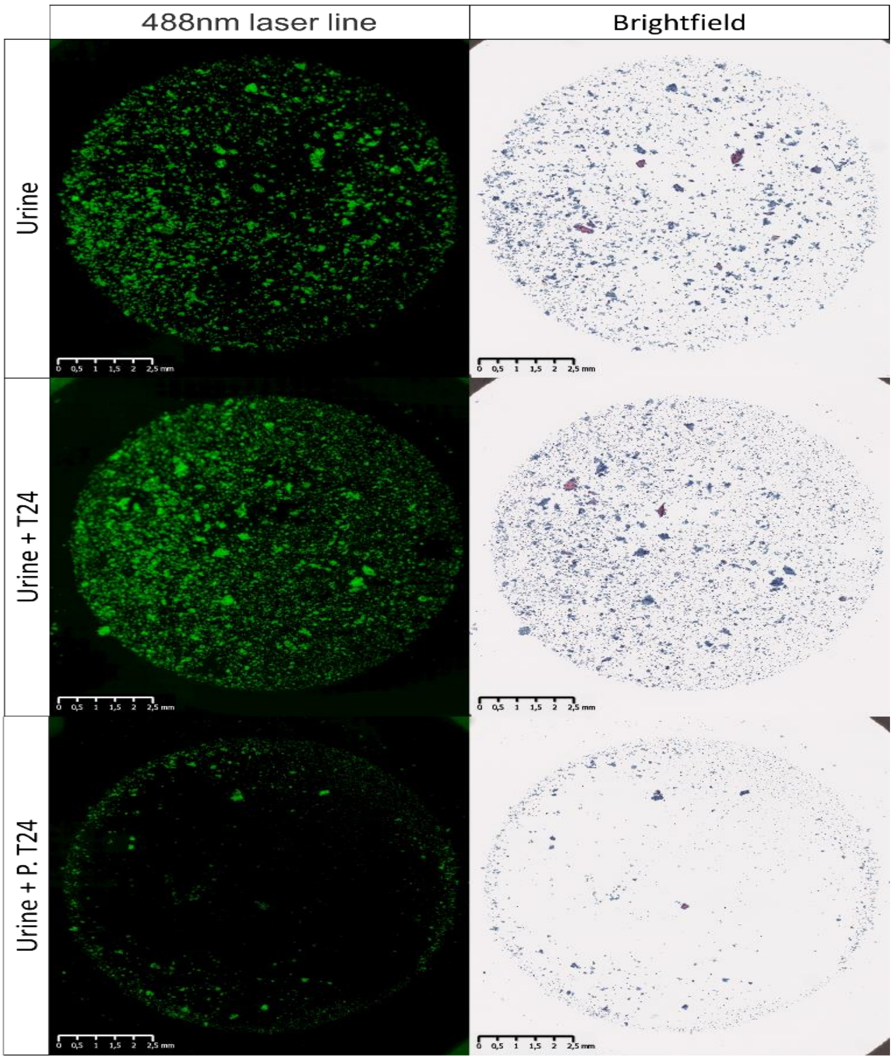

Figure S4. Fluorescence and PMF of UCU and UCUT. Global view of images acquired on scanner of healthy urine alone (Urine), supplemented with T24 (Urine + T24) or supplemented with T24 permeabilized (Urine + P. T24) with 0.2% triton for 90 seconds

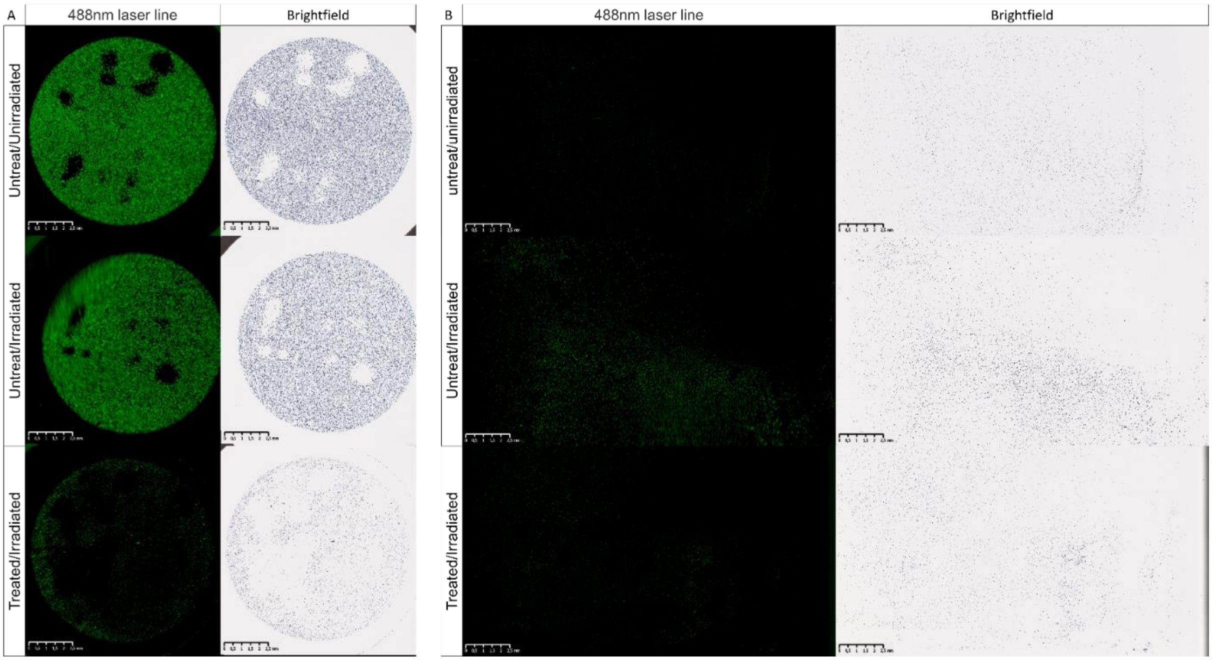

Figure S5. Involvement of senescence in the modulation of PMF. Global view of images acquired on scanner of the effect of irradiation with or without treatment after nine days on a) the PMF of MRC-5hTERT; b) the PMF of T24

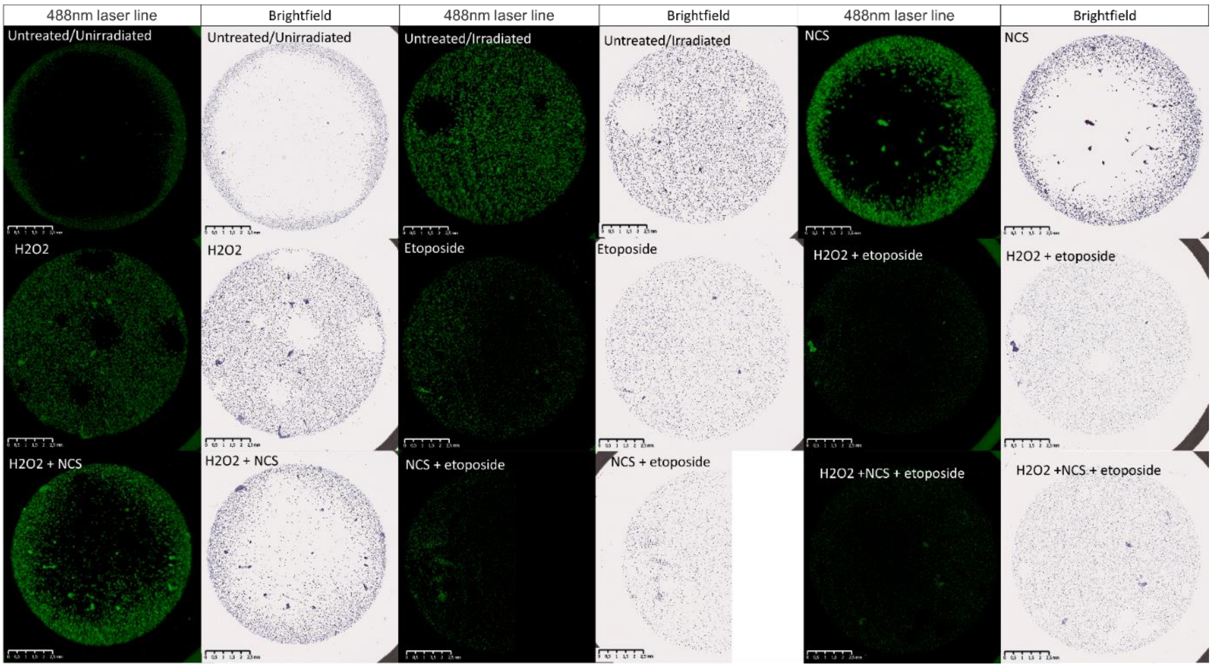

Figure S6. Involvement of the treatment on the modulation of PMF. Global view of images acquired on scanner of the effect of the different treatments and molecules to induce senescence on the T24 PMF after nine days.

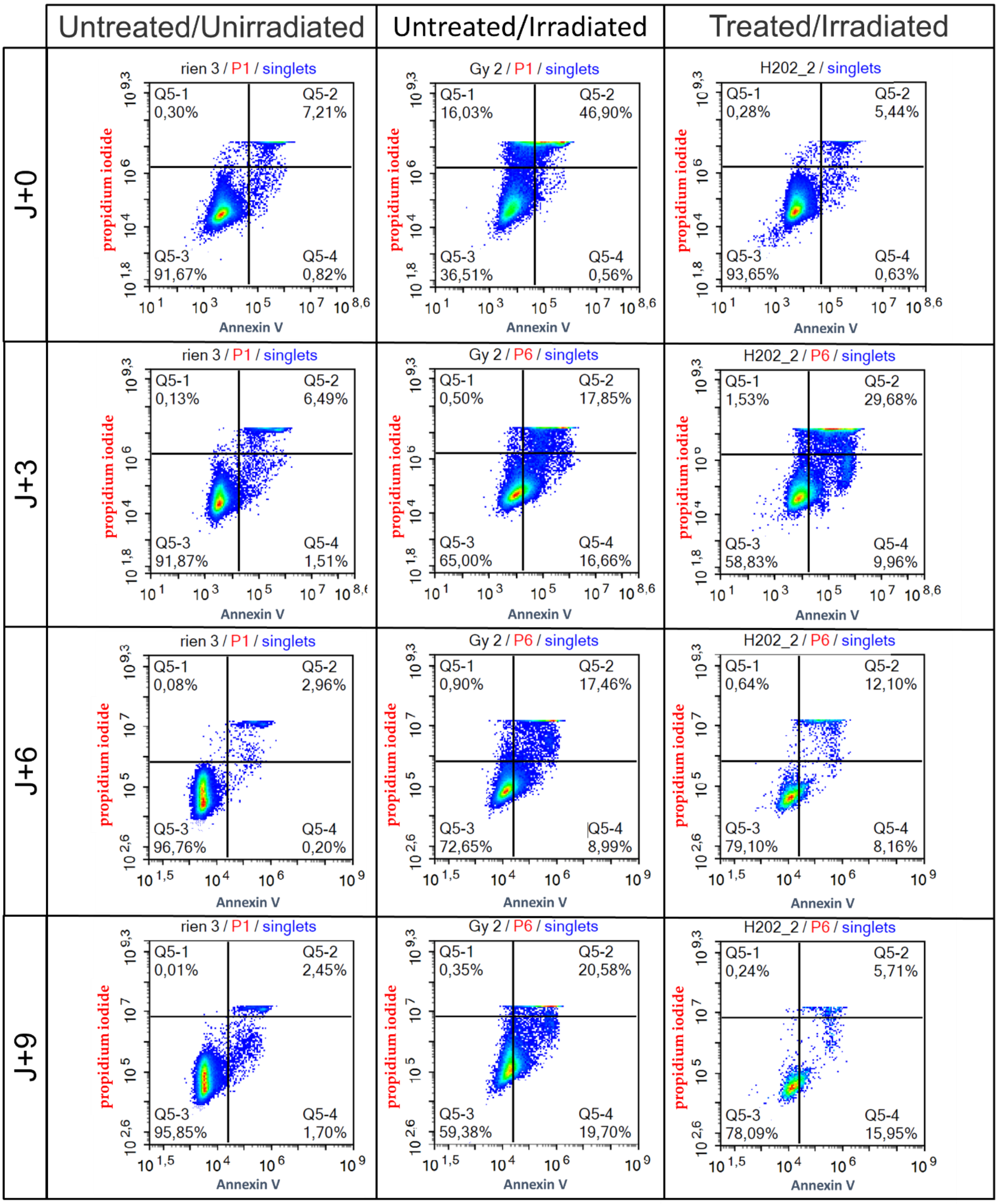

Figure S7. Total cell death determination by flow cytometer after induction of cellular stress by irradiation and treatment on T24. On graph: bottom right square: early apoptosis, top right square: late apoptosis, top left square: necrosis. Cells were labelled with annexin V and propidium iodide.

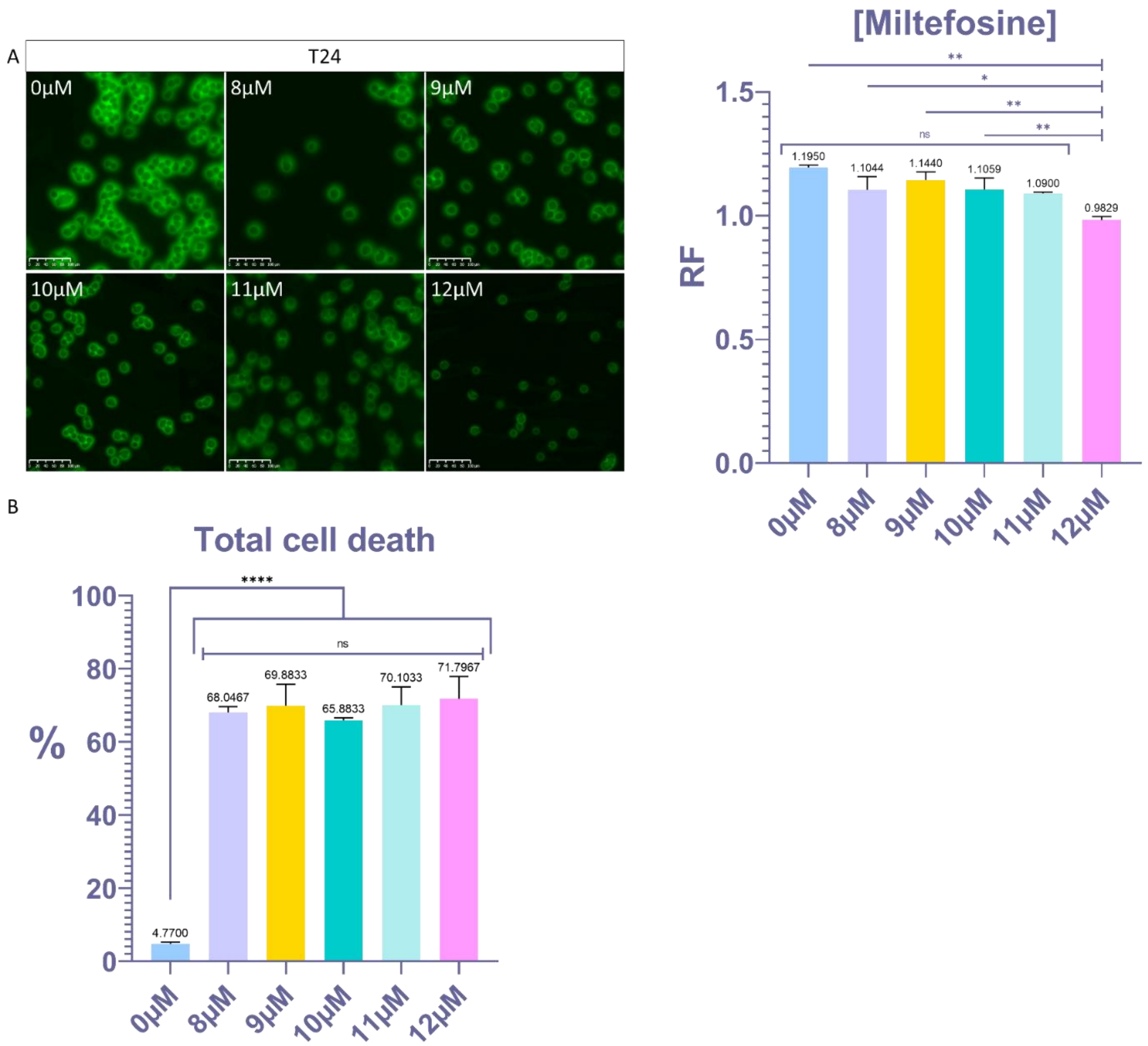

Figure S8. Disruption of the plasma membrane by miltefosine treatment. a) Images acquired on scanner of T24 after 24h treatment with different concentrations of miltefosine and quantification of their PMF b) Flow cytometer determination of early apoptosis, late apoptosis and necrosis combined after 24h treatment with different concentrations of miltefosine, annexin V and propidium iodide labelling. Bar graphs represent mean (SD) (ns > 0.05; ; ** P < 0.1) ** P < 0.01; **** P < 0.0001).

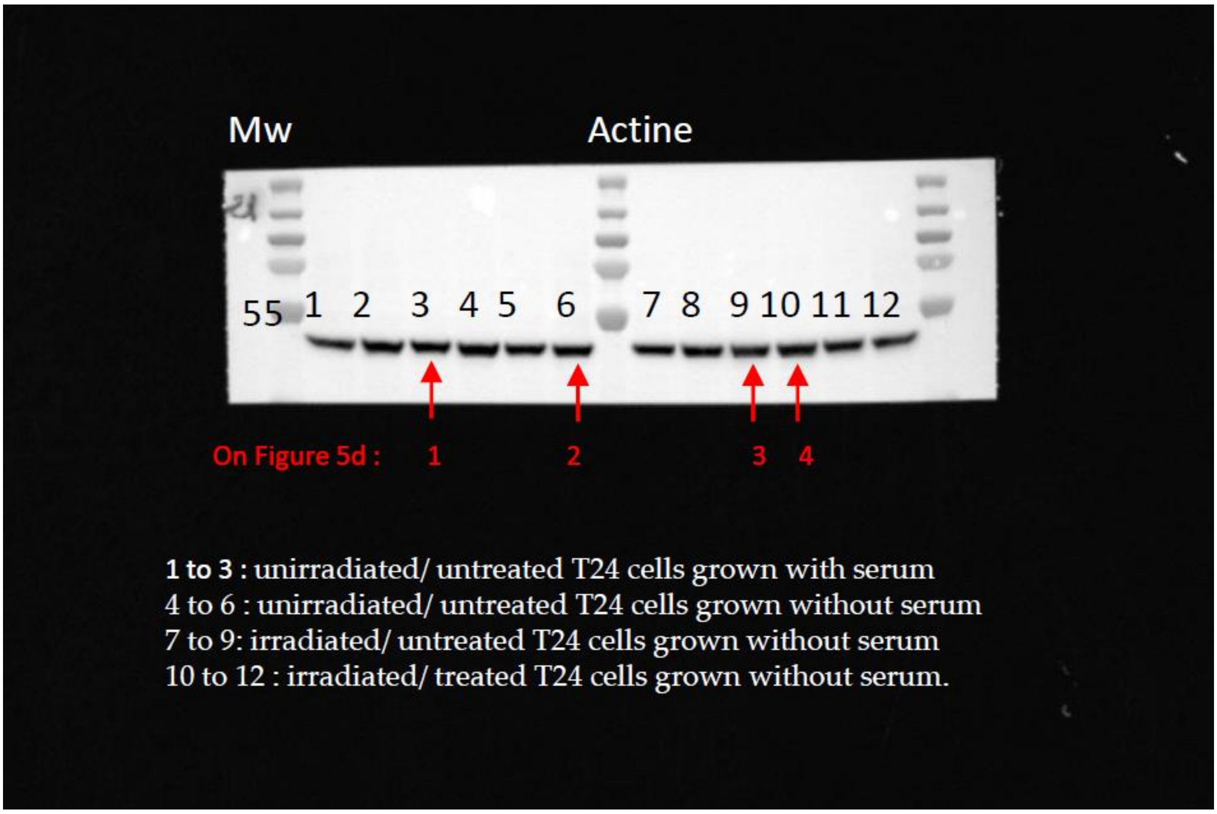

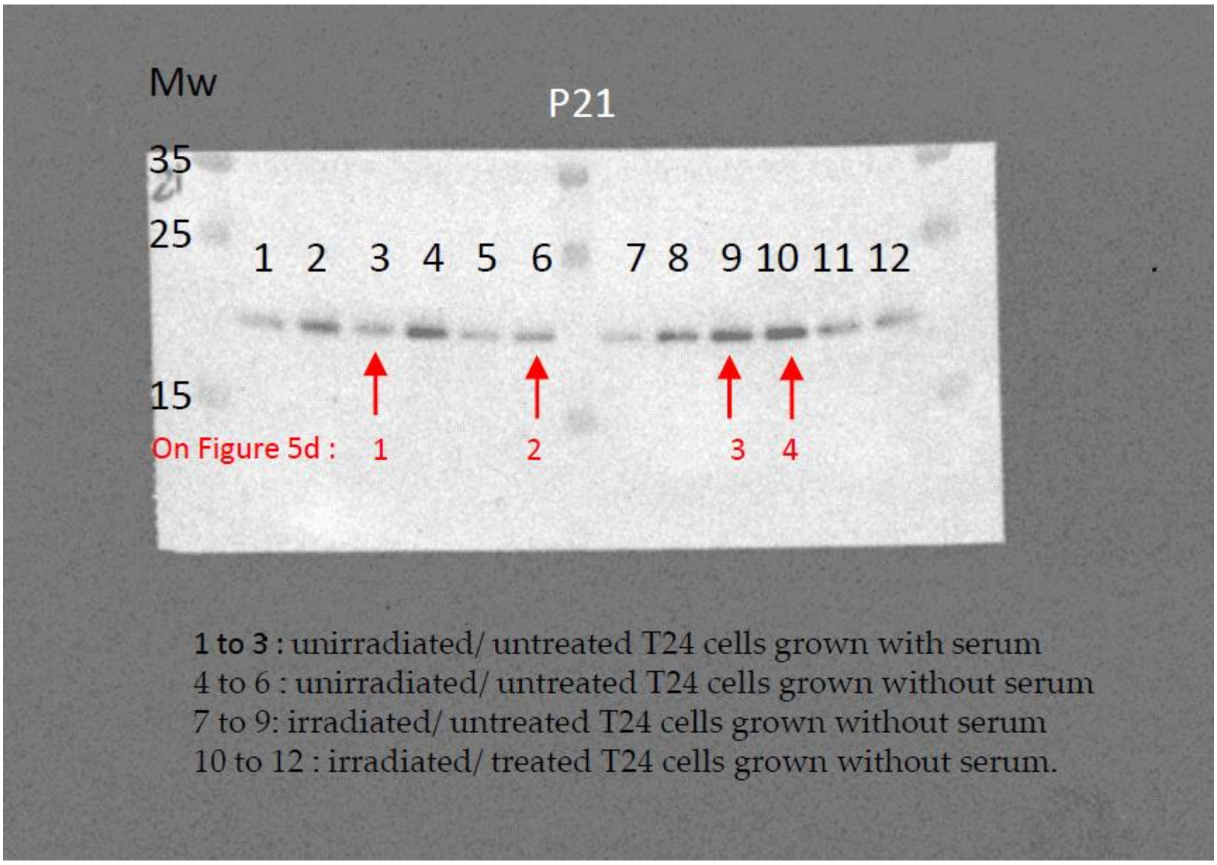

Figure S9. Original blots of Figure 5D.
